# High-intensity focused ultrasound for the treatment of fibroadenomata (HIFU-F) study

**DOI:** 10.1186/s40349-015-0027-6

**Published:** 2015-04-14

**Authors:** Mirjam C L Peek, Muneer Ahmed, Michael Douek

**Affiliations:** Research Oncology, Division of Cancer Studies, King’s College London, Guy’s Hospital Campus, Great Maze Pond, London, SE1 9RT Great Britain UK

**Keywords:** Ultrasound-guided high-intensity focused ultrasound (HIFU), Benign breast disease, Fibroadenoma, Non-invasive treatment

## Abstract

**Background:**

Breast fibroadenomata (FAD) are the most common benign lesions in women. For palpable lesions, there are currently three standard treatment options: reassurance (with or without follow-up), vacuum-assisted mammotomy (VAM) or surgical excision. High-intensity focused ultrasound (HIFU) ablation has been used in the treatment of FAD. The drawback of HIFU is its prolonged treatment duration. The aim of this trial is to evaluate circumferential HIFU treatment for the effective ablation of FAD with a reduced treatment time.

**Methods/design:**

Fifty patients (age ≥18 years) will be recruited with symptomatic FAD, visible on ultrasound (US, grade U2 benign). In patients ≥25 years, cytology or histology will be performed to confirm the diagnosis of a FAD. These patients will receive HIFU treatment using the US-guided Echopulse device (Theraclion Ltd., Malakoff, France) under local anaesthesia. An additional 50 patients will be recruited and contacted 6 months after discharge from the breast clinic. These patients will be offered an US scan to determine the change in size of their FAD. This natural change in size will be compared to the decrease in size after HIFU treatment. Secondary outcome measures include post-treatment complications, patient recorded outcome measures, mean treatment time and cost analysis.

**Trial registration:**

Current Controlled Trials: ISRCTN76622747.

## Background

Fibroadenomata (FAD) are the most common benign breast lesions in women. FAD can occur at any age but are most common between 20 and 30 years of age. FAD occur in about 10% of all woman and account for about 50% of all breast biopsies performed [1]. Studies revealed that up to 59% of FAD will show regression or complete resolution within 5 years [1]. Malignant transformation within FAD is considered exceptionally rare (0.002%–0.0125%) and there is a 1.3–2.1 increased risk of breast cancer in women with FAD compared to the general population [2]. FAD are encapsulated from their surrounding tissues and can be considered as an aberration of normal breast development rather than a true neoplasm. On histology, FAD consist of combined proliferation of epithelial and fibroblastic tissue elements which are slowly growing and oestrogen dependent [3,4].

The diagnosis of a FAD can be made by standard triple assessment (clinical examination, imaging and histopathology). During clinical examination, 50%–67% of breast lesions identified as a FAD are confirmed as FAD on imaging and/or histopathology due to similar characteristics with other benign conditions [5,4]. Therefore, more accurate diagnostic methods are required to get the correct diagnosis. Ultrasound (US) is the main diagnostic imaging modality used for the diagnosis of FAD [5], and fine needle aspiration cytology (FNAC) or core needle biopsy (CNB) can be used to get tissue confirmation of the diagnosis. The overall diagnostic accuracy of this triple assessment is approximately 70%–80%, but an accurate differentiation between a benign and a malignant lesion is provided in 95% [5,4].

The management of non-palpable FAD is conservative with reassurance. For palpable FAD, there are currently three main treatment options available: reassurance (with or without follow-up), vacuum-assisted mammotomy (VAM) and surgical excision. VAM is not officially licensed for the treatment of FAD only to obtain the diagnosis of a lesion. Intervention is normally offered to patients with large FAD or rapidly growing lesions or to patients requesting excision of the lesion due to anxiety, discomfort or breast distortion [4]. In other cases, surgical removal can cause scarring and poor cosmetic outcome [5,6].

A new technique in the treatment of FAD is high-intensity focused ultrasound (HIFU) ablation. This is a non-invasive ablative technique in which the FAD is treated with focused consecutive repeated US pulses, while surrounding tissues are protected from the applied heat.

The basis of HIFU therapy is a HIFU pulse of several seconds generated by a piezoelectric US transducer. The US field is insonated via a coupling media overlaying the tissue to the targeted area. Due to the high local concentration of acoustic energy in the focal spot, the tissue in a small volume is heated rapidly, and a sharp circumscribed lesion caused by thermal coagulation will be induced [7]. HIFU has been clinically applied for the treatment of invasive breast cancer and demonstrated coagulative necrosis on pathological assessment, regression of tumour size and loss of proliferative activity with a minimal side effect profile [8-16]. Pathological examination with Victoria Blue and Ponceau histochemical staining has been used to assess tumour vascular wall destruction and immunohistochemical staining for proliferative markers using biotin-streptavidin-peroxidase [13]. Cell viability has been determined by staining for active dehydrogenase using 2,3,5-triphenyltetrazolium chloride (TTC) [14].

The current limitation of HIFU is the prolonged treatment duration associated with the procedure. Since a single pulse generates a rather small tissue lesion, a lot of these consecutive repeated pulses have to be applied with an adequate idle time in between to prevent overheating and damage to normal tissue [7]. We propose to overcome the prolonged treatment duration by applying HIFU pulses to the circumferential surface area of the lesion rather than their whole volume. We propose to perform this upon a cohort of patients with benign breast tumours in the form of FAD using the US-guided Echopulse^TM^ (Theraclion Ltd., Malakoff, France) HIFU system, which is CE marked for the treatment of breast FAD.

## Methods/design

### Patients

We will undertake treatment on 50 patients with confirmed FAD. Patients will be identified in three ways: (1) at multidisciplinary meetings (MDM), where all patients who undergo CNB or FNAC are discussed; (2) patients scheduled for surgical excision of a FAD; and (3) patients visiting the Breast Clinic requesting surgical excision. All patients will be approached in the Breast Clinic or via telephone asking if they are interested in receiving a patient information sheet on the HIFU-F trial which describes the procedure in detail. If patients are interested, a second telephone call will be made to check if the patient would like to participate in the study and to answer any questions. Patients who are willing to participate in the study will provide informed written consent on the day of elective surgery or at a prior hospital visit (during pre-assessment). Details of all patients approached about the trial will be recorded on the patient-screening log and kept in the Investigator Site File.

### Inclusion and exclusion criteria

**Figure 1:**
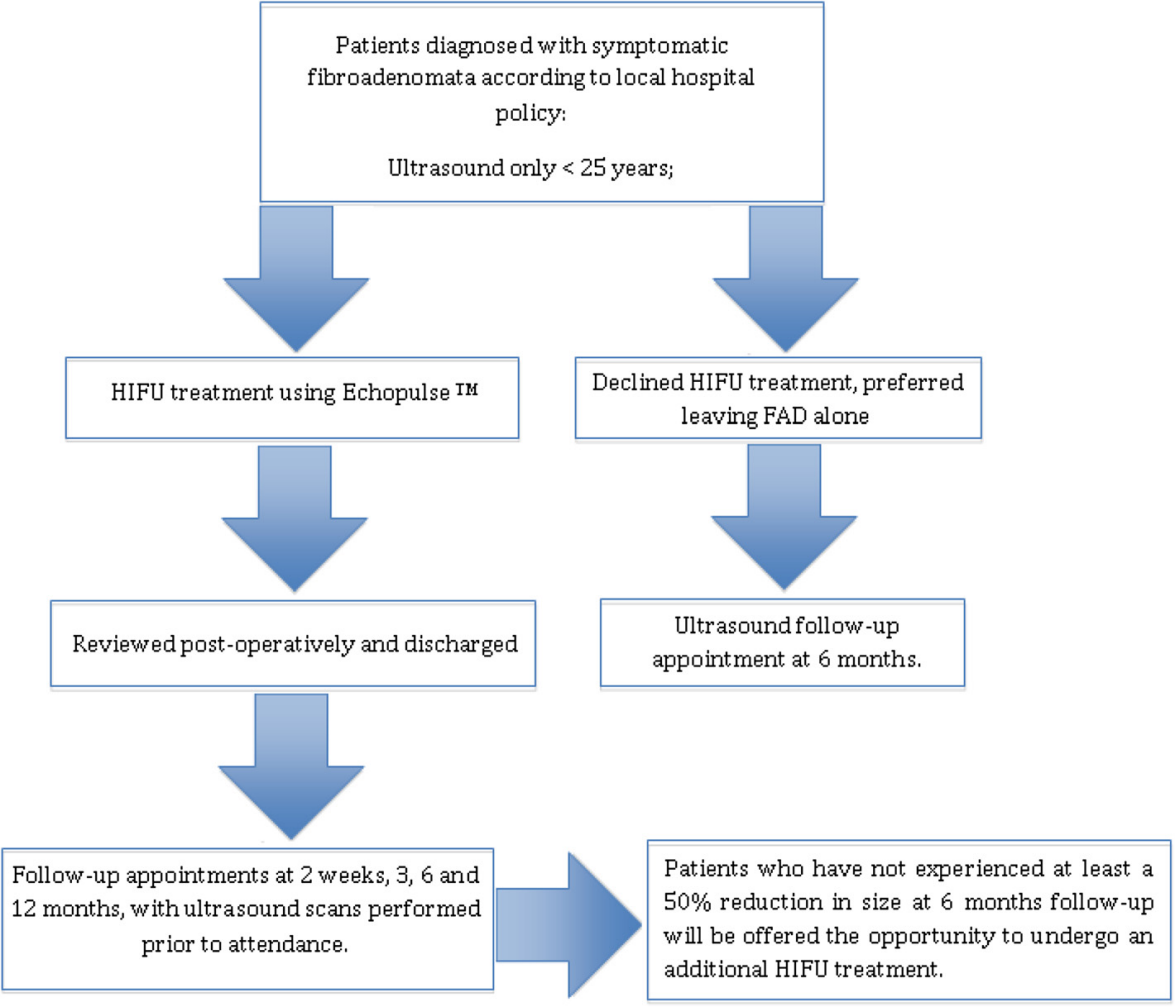
HIFU-F trial scheme.

Patients eligible for this trial are patients ≥18 years of age with FAD diagnosed according to local hospital protocol visible on US (graded U2 benign). In patients ≥25 years of age, confirmation is required by either FNAC (C2) or CNB (B2) (Figure 1). The definitive diagnosis of FAD must be confirmed by the breast MDM.

Exclusion criteria are FAD with atypia or suspicion of phyllodes (graded B3/C3 or greater), pregnant or lactating women, women with breast implants and women with a history of laser or radiation therapy to the targeted breast.

### HIFU treatment group

All patients in the HIFU treatment group will attend the HIFU treatment using the US-guided Echopulse device (Theraclion Ltd., Malakoff, France) as a day-case procedure to be performed in the Clinical Research Facility (CRF) at Guy’s Hospital. The breast lesions will be ablated real-time under US guidance using a 7.5–12-MHz diagnostic US transducer (Theraclion Ltd., Malakoff, France). This device produces therapeutic US energy by a 56 ± 1 mm diameter 3.0-MHz imaging transducer with a central hole of 11 ± 1 mm for the coaxial imaging transducer. The transducer ablates a tissue volume of approximately 9 mm in length and 2 mm in width.

Patients will be placed in a supine position and degassed local anaesthetic will be administered subcutaneously under US guidance. The breast will be immobilised when necessary with an immobilisation system (Theraclion Ltd., Malakoff, France). A coupling media in the form of a gel pad attached to the device will be lowered onto the treatment site. The Echopulse™ will be used to image the lesion in two perpendicular dimensions and set to deliver HIFU to the circumferential surface area of the lesion by deselecting the centre of the lesion. A laser pointer detects any significant movement from the patient and pauses the treatment. Treatment times at beginning (first pulse administered) and end (last pulse administered) of the treatment will be recorded. Actual treatment time will be compared to the treatment time required for complete whole-lesion ablation. Once the treatment is complete, the patient will be observed in the discharge suite before being discharged. All patients will be reviewed post-treatment at 2 weeks and 3, 6 and 12 months with a repeat US scan at each appointment.

Patients who have not experienced at least a 50% reduction in volume of their FAD at 6 months follow-up on US will be offered the opportunity to undergo an additional HIFU treatment.

### Control group

Another 50 patients with symptomatic palpable FAD who have been discharged from the Breast Clinic since the HIFU-F trial started (12 December 2013) will be recruited. These patients will be contacted 6 months after their discharge and will be offered an US scan in order to determine the change in volume of their FAD.

This cohort will be selected in order to identify the natural course of FAD over a period of 6 months from diagnosis. Analysis will be performed to compare the difference in volume between the HIFU-treatment group *versus* control group initially when 20 control patients have been recruited.

### Outcome measures

The primary outcome measure is the change in volume of FAD as recorded by US. Secondary outcome measures will include complications, patient-recorded outcome measures, mean treatment time and cost-effectiveness of the HIFU treatment compared to surgical excision.

All patients will be asked prior to receiving HIFU treatment and again at 6 and 12 months post-treatment to submit sections of the Breast-Q Breast Conserving Therapy Modules to assess patient-recorded outcome measures.

A two-sample *T*-test will be performed to determine if there is a significant difference in FAD volume reduction between the study group and the control group. To determine if the variances are equal or unequal, we will perform a two-sample *F*-test. A two-sample *T*-test will also be used to determine if there is a significant reduction in lesion volume at 6 months compared to prior to treatment.

## Discussion

At our unit, over 600 FAD were identified on US imaging in 2012 of which 6 underwent VAM and 60 formal surgical excision. Difficulties could be encountered during recruitment of patients from the MDM. Clinicians could be hesitant to allow recruitment of patients who have symptomatic FAD, but would not generally be offered intervention. Patients offered surgical excision are usually patients with atypical FAD, lesions with a suspicion of phyllodes tumour and fast growing and large FAD. Most of these patients are not eligible for HIFU treatment and should be offered surgical excision. Large lesions are not an exclusion criteria, but HIFU treatment would take significantly longer and multiple treatments might be needed to treat the whole FAD.

In general, FNAC and CNB could be performed in all patients; however, local protocol permits women <25 years of age in which US and physical examination reveals a solid lesion with typical benign characteristics to omit histopathology. This is due to the low incidence of breast cancer in woman of this age group [17,18].

The primary outcome measure is the volume decrease of the FAD as visible on US. US is the standard diagnostic imaging modality for FAD and accessible to patients and staff. Furthermore, the FAD must be visible on US to be eligible for HIFU; it should therefore be possible to visualise and measure the FAD post-treatment as well. Disadvantages of US as an imaging modality are the inter-observer variability between the measurements performed by different ultrasonographers. The ideal imaging modality would be accessible and overcome inter-observer variability.

In this protocol, the diameters of the FAD are measured in three dimensions to calculate the volume of the FAD with formula (1), in which A, B and C are the largest diameters in the three dimensions.

1 $$ V\kern0.5em =\kern0.5em \frac{4}{3}\kern0.5em \times \kern0.5em \pi \kern0.5em \times \kern0.5em \left(\frac{1}{2}A\right)\kern0.5em \times \kern0.5em \left(\frac{1}{2}B\right)\kern0.5em \times \kern0.5em \left(\frac{1}{2}C\right) $$

Measuring one diameter is not sufficient since the decrease enough since the decrease in size could be different in the different dimensions, and with the volume, these are all taken into account.

The disadvantage of this trial is that no histology or cytology is obtained post-treatment. Only in patients opting for surgical excisions after HIFU treatment could the histology be obtained. Histopathology could provide more accurate information about the changes in FAD size and surrounding tissues over time. CNB or FNAC could be performed to obtain this as well; however, these would likely hamper recruitment.

## Abbreviations

CRF: Clinical Research Facility
FAD: Fibroadenoma
HIFU: High-intensity focused ultrasound
MDM: Multidisciplinary meeting
TTC: 2,3,5-triphenyltetrazolium chloride
US: Ultrasound
VAM: Vacuum-assisted mammotomy.
